# A Feasibility Study to Evaluate Changes in Urinary Metabolites after OnabotulinumtoxinA Injection for Refractory Overactive Bladder

**DOI:** 10.3390/metabo12090880

**Published:** 2022-09-19

**Authors:** Laura M. Tellechea, Samantha Freeman, Ilir Agalliu, Melissa A. Laudano, Sylvia O. Suadicani, Nitya Abraham

**Affiliations:** 1 Department of Obstetrics & Gynecology, Montefiore Medical Center, Albert Einstein College of Medicine, Bronx, NY 10461, USA; 2 Department of Urology, Montefiore Medical Center, Albert Einstein College of Medicine, Bronx, NY 10461, USA; 3 Department of Epidemiology and Population Health, Albert Einstein College of Medicine, Bronx, NY 10461, USA

**Keywords:** overactive bladder, urinary metabolome, biomarkers, onabotulinumtoxinA

## Abstract

Metabolomics analysis of urine before and after overactive bladder (OAB) treatment may demonstrate a unique molecular profile, allowing predictions of responses to treatment. This feasibility study aimed to correlate changes in urinary metabolome with changes in OAB symptoms after intravesical onabotulinumtoxinA (BTX-A) injections for refractory OAB. Women 18 years or older with non-neurogenic refractory OAB were recruited to complete OAB-V8 questionnaires and submit urine samples before and after 100 units intravesical BTX-A injection. Samples were submitted to CE-TOFMS metabolomics profiling. Data were expressed as percent of change from pre-treatment and were correlated with OAB-V8 score improvement. Urinary metabolite changes in the OAB-V8 groups were compared using the Kruskal–Wallis test, and associations between metabolites and OAB-V8 scores were examined using quantile regression analysis. Of 61 urinary metabolites commonly detected before and after BTX-A, there was a statistically significant decrease in adenosine and an increase in N8-acetylspermidine and guanidinoacetic acid levels associated with OAB score improvement, suggesting that intravesical BTX-A injection modifies the urinary metabolome. These urinary metabolites could provide insight into OAB pathophysiology and help identify patients who would benefit most from chemodenervation.

## 1. Introduction

An overactive bladder (OAB) is characterized by urinary urgency, usually with frequency and nocturia, with or without urgency urinary incontinence [1]. Most women without known neurologic conditions are classified as having “idiopathic OAB” and are treated with a “one size fits all” approach that progresses from conservative to invasive therapies based on the patient’s preference and the physician’s experience [2]. The reality is that OAB is multifactorial in etiology and may benefit from a more personalized approach to treatment.

Classification of OAB by etiology is limited by our current diagnostic tools. Urodynamics is invasive and may not always demonstrate detrusor overactivity [3]. Surveys focus on OAB symptoms and severity but not etiology. Changes in urinary levels of urothelial signaling molecules (e.g., adenosine triphosphate (ATP), NGF (nerve growth factor), nitric oxide (NO)) and metabolites have been used in pre-clinical studies to indirectly assess changes in bladder function and gain insights into bladder pathophysiology [4].

Metabolomics, the large-scale analysis of metabolites in biological systems, has been promising during exploration of potential biomarkers in pathological conditions, such as bladder cancer, metabolic syndrome, and interstitial cystitis [5,6,7,8]. Metabolomics has also been applied in OAB studies. For example, a study comparing the urinary metabolome of female patients with OAB and healthy controls found that metabolites involved with mitochondrial dysfunction, ketosis, and oxidative stress were associated with OAB severity [9]. However, changes in the urinary metabolome after OAB treatment have not been evaluated. Most importantly, it remains to be determined whether changes in urinary metabolites observed post-treatment correlate with OAB symptom improvement.

Our study objective was to assess the feasibility of characterizing the urinary metabolome and comparing changes in the metabolome before and after intravesical onabotulinumtoxinA (BTX-A), which is a treatment for women with non-neurogenic refractory OAB.

Identifying urinary metabolites that may correlate with OAB symptom improvement after chemodenervation could guide our counseling of patients toward treatments with a higher chance of success, instead of using a “one-size fits all” approach. Furthermore, evaluating changes in the urinary metabolome after treatment may provide unique insights into OAB pathophysiology.

## 2. Materials and Methods

### 2.1. Study Design

We conducted a prospective, observational feasibility study among women with refractory OAB at our urban academic pelvic floor clinic (Montefiore Medical Center, Bronx, NY, USA) who were presenting for an intravesical BTX-A injection from June 2019 through March 2020. Women were recruited and consented on the day of BTX-A injection. Urine samples were collected prior to BTX-A injection and at every post-injection follow-up visit. Urine samples were aliquoted into 1 mL tubes, immediately frozen on dry ice, and kept at −80 °C until analysis. All metabolomic results were corrected for creatinine. The OAB-V8 questionnaire was administered at the time of urine collection to assess OAB symptom severity. The study protocol was approved by the Institutional Review Board of the Albert Einstein College of Medicine (IRB number: 2019-10009), Bronx, NY, USA.

### 2.2. Participants

Women aged 18 years or older who failed first- and second-line OAB treatment and opted for third-line treatment with intravesical BTX-A injections were recruited for this study. All patients were on antibiotics for urinary tract infection (UTI) prophylaxis and underwent a point-of-care urinalysis at the time of BTX-A injection. Patients with neurogenic lower urinary tract dysfunction, spinal cord injury, urinary retention, or any condition requiring intermittent catheterization, malignancy or history of pelvic radiotherapy, concomitant bladder stones, or pelvic organ prolapse > stage 2 in the anterior or apical compartment, were bed-bound or wheelchair-bound, pregnant, or had difficulty completing a survey due to literacy or language were excluded. Post-void residual (PVR) volumes were collected at every follow-up visit, and patients were excluded if their PVR was above 200 cc. 

### 2.3. Intravesical BTX-A Injection

BTX-A injection was performed as an outpatient office procedure. The patient was catheterized to empty the bladder and a solution of 2% lidocaine was instilled for 20–30 min prior to injection. One hundred units of BTX-A (BOTOX^®^, onabotulinumtoxinA, Allergan, Irvine, CA, USA) were diluted in 10 mL of normal saline [10]. The BTX-A injection was performed using a 17 French flexible cystoscope (Olympus, Breinigsville, PA, USA) and a 70 cm 6 French injection needle (Injetak, Laborie, Portsmouth, NH, USA) into 10 sites of the bladder wall, including the bladder base and the trigone.

### 2.4. Overactive Bladder Symptom Score (OAB-V8)

The OAB-V8 symptom scale is an 8-item questionnaire adapted from the OAB-q assessment tool. It has demonstrated predictive validity in diagnosing and evaluating treatment for OAB [11]. Clinical evaluation and a score of 8 or above on the OAB-V8 scale were used to diagnose OAB [12]. OAB-V8 questionnaires were administered prior to BTX-A treatment and at every follow-up visit at the time of urine collection. Surveys were available in both English and Spanish.

Following BTX-A injections, patients were classified as either responders or non-responders based on the change in their OAB-V8 score. A patient was considered a responder to BTX-A treatment when an improvement of 8 points or more was observed on the OAB-V8 questionnaire, whereas non-responders were those with a change in OAB-V8 score that was less than 8. Responders were further categorized as follows: 8 to 15-point improvement = mild response; 16 to 23 = moderate response, and ≥ 24 = marked response. Cut-off values were separated by 8 points, which are multiples of the first cut-off value screening for the presence of OAB [11].

### 2.5. Urinary Metabolome Analysis

Metabolomics profiling was conducted at Human Metabolome Technologies (HMT; Tsuruoka, Japan). Briefly, 20 µL urine samples were mixed with 20 µL of Milli-Q water containing internal standards (1 mM) and 60 µL of Milli-Q. The mixture was filtrated through a 5 kDa cut-off filter (ULTRAFREE-MC-PLHCC, HMT) to remove macromolecules. CE-TOFMS (capillary electrophoresis time of flight mass spectrometry) based metabolome analysis was performed in two modes for cationic and anionic metabolites, following routine protocols.

Measurement was carried out using an Agilent CE-TOFMS system (Agilent Technologies, Waldbronn, Germany) controlled by Agilent G2201AA ChemStation software version B.03.01 for CE (Agilent Technologies). The metabolites were separated using a fused-silica capillary (50 μm i.d. × 80 cm total length) with commercial electrophoresis buffer (H3301-1001 for cation analysis and H3302-1021 for anion analysis, HMT). The sample was injected at a pressure of 50 mbar for 10 s (approximately 10 nL) in cation analysis and 22 s (approximately 25 nL) in anion analysis. The MS scanned from *m*/*z* 50 to 1000. Other conditions were as described previously [13,14,15]. Over 1000 metabolites were analyzed within this basic scan.

Peaks were extracted using the MasterHands automatic integration software (ver. 2.17.1.11, Keio University, Tsuruoka, Japan) to obtain information including *m*/*z*, migration time (MT), and peak area values [16]. Peaks were then annotated with putative metabolites assigned from the HMT’s standard library and Known–Unknown peak library, based on *m*/*z* and MT values. The tolerance range for peak annotation was ±10 ppm for *m*/*z* and ±0.5 min for MT. In addition, peak areas were normalized against those of the internal standards. The values were further normalized to the creatinine levels to account for urine dilution, sample volume effects, and kidney function.

### 2.6. Statistical Analysis

We compared demographic and lifestyle characteristics, as well as comorbid conditions among patients, in the three responder and one non-responder BTX-A treatment groups. We achieved this using one-way analysis of variance (ANOVA), or Kruskal–Wallis test (for continuous normally or non-normally distributed variables, respectively), and chi-square tests (for categorical variables). All tests were 2-sided, with *p* < 0.05 indicating statistical significance. Urinary metabolites were expressed as percent change from baseline pre-BTX-A values and were calculated by subtracting the post-BTX-A from the pre-BTX-A levels, then dividing this value by the pre-BTX-A level (values were multiplied by 100 to express as a percentage). Since the urinary metabolites were not normally distributed, the Kruskal–Wallis test and quantile regression analysis were used to compare urinary metabolite levels across changes in the OAB-V8 score category (these methods do not require normality assumptions). We also examined associations between changes in urinary metabolites with OAB groups using quantile regression analysis, adjusting for age, Hispanic ethnicity, BMI, and DM. The comparison group in these regression models were women who did not respond to treatment (non-responders). Correlations among age, BMI, and different urinary biomarkers were evaluated using Spearman’s correlation coefficients. All statistical data analyses were carried out in STATA v.17 (StataCorp LLC, College Station, TX, USA).

## 3. Results

### 3.1. Patient Demographics

**Figure 1:**
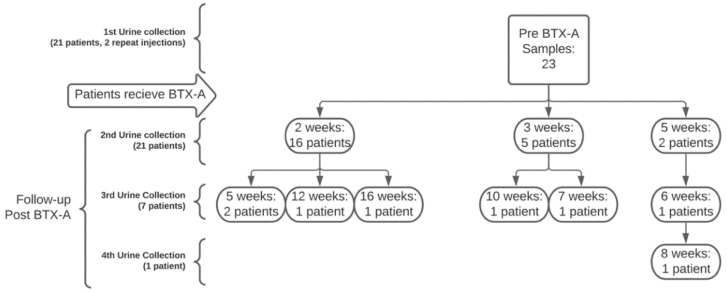
Patient flowchart with time of initial recruitment, BTX-A injection, and follow up.

A total of 21 patients were eligible and consented to participate in this study. The average age was 56.8 (SD = 10.9) years, and the average BMI was 31.1 kg/m^2^ (SD = 4.9). The majority of patients were Hispanic (76.2%). One patient was a smoker, and three had diabetes (Table 1). Of the patients, 61% had severe OAB, as defined by a pretreatment OAB-V8 score of 30 or higher, and 22% and 17% had moderate and mild OAB, respectively. Although women were scheduled for follow-up between 2 and 6 weeks, the actual follow-up time was variable (Figure 1). Two women underwent a repeat injection, resulting in 23 samples collected pre-BTX-A. The total number of post-injection samples collected was 31. No injection-related complications or toxin-related side effects were observed. Of the 31 post-injection samples, 23 were responders and 8 were non-responders, resulting in a 67% OAB improvement rate (Table 1). There were no statistically significant differences in demographics and lifestyle variables when comparing patients by OAB improvement group.

### 3.2. Metabolites

**Figure 2:**
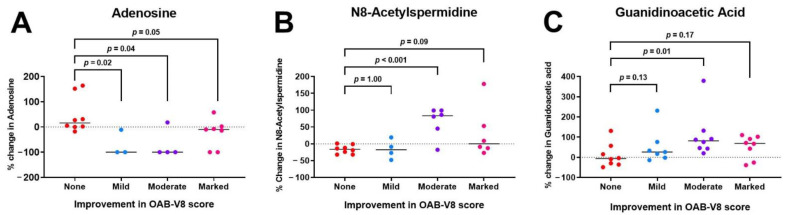
Percentage of change in urinary metabolite levels by improvement in OAB-V8 score: None (no improvement) < 8-point change, Mild = 8–15-point change, Moderate = 16–23-point change, Marked ≥ 24-point change. *p* values were generated by comparing the Mild, Moderate, and Marked groups to the None group as shown in the figure.

From CE-TOFMS measurement, 329 peaks were detected and annotated on the basis of HMT’s standard library and Known–Unknown peak library. Of 61 target metabolites detected and quantified, 3 (4.9%) metabolites were shown to significantly change in patients that responded to BTX-A treatment when compared with non-responders (Figure 2). Except for guanidinoacetic acid, these changes persisted after adjusting for potential confounders (Table 2).

#### 3.2.1. Adenosine

Urinary adenosine levels decreased significantly in patients who reported improvement in OAB symptoms following intravesical BTX-A injections when compared with non-responders (overall *p* = 0.031; Figure 2A). The median decrease was 100% for patients in both mild (*p* = 0.02) and moderate (*p* = 0.04) OAB-V8 improvement groups, but only 10% (*p* = 0.055) for patients with marked OAB-V8 improvement (Figure 2A). Results were similar after adjusting for other covariates, showing a more significant decrease in the mild and moderate OAB-V8 improvement groups, but not in the marked category (Table 2; adjusted models).

#### 3.2.2. N8-Acetylspermidine

**Figure 3:**
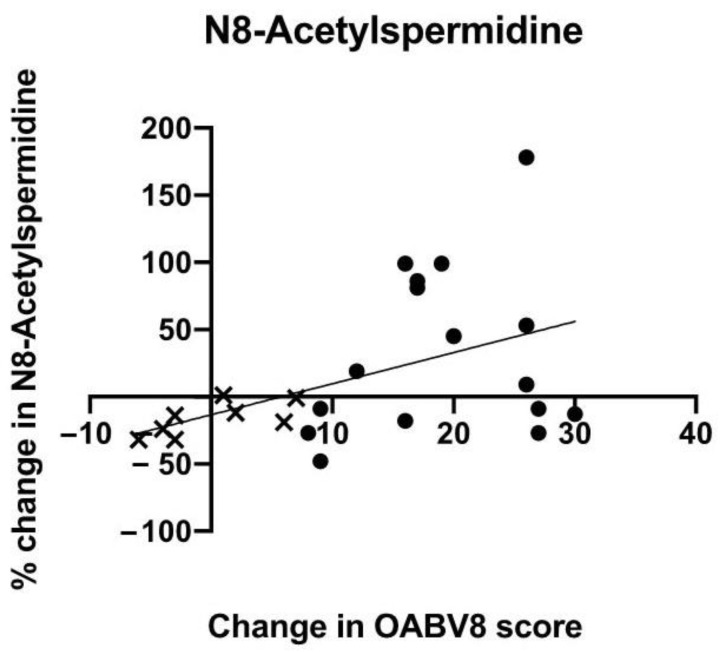
Median regression model showing that the percent change in urinary N8-acetylspermidine levels of patients in the non-responder (×) and responder (●) groups were directly correlated with changes in OAB-V8 scores after adjusting for age, BMI, diabetes mellitus, and race/ethnicity. β-coefficient slope = 2.80 (95% CI 0.04, 5.55), *p* = 0.047.

Urinary N8-acetylspermidine levels increased significantly in patients reporting improvement in OAB symptoms following intravesical BTX-A injections when compared with non-responders (overall Kruskal–Wallis *p* = 0.032; Figure 2B). However, the only statistically significant increase was observed in the moderate OAB improvement group (83.5%; *p* < 0.001) when compared with non-responders (Figure 2B). In the median regression model, the percent change was shown to directly correlate with changes in the OAB-V8 scores (β = 2.80, 95% CI 0.04–5.55: Figure 3).

#### 3.2.3. Guanidinoacetic Acid

Urinary guanidinoacetic acid levels significantly increased in the moderate OAB-V8 improvement group when compared with non-responders (Figure 2C). The average increase in this group was 81.5% (*p* = 0.01; Figure 2C), but this difference was attenuated to 47% after adjusting for covariates and was no longer statistically significant (Table 2).

## 4. Discussion

This is a novel feasibility study that compared urinary metabolite levels before and after intravesical BTX-A injections in women with refractory non-neurogenic OAB and correlated changes with patients’ reported improvement in OAB symptoms. In this study, we observed significant changes in certain urinary metabolites after intravesical BTX-A injections.

Urinary levels of adenosine were significantly decreased in patients who responded to BTX-A treatment compared to levels in patients without reported improvement. Adenosine is a purine derived from the breakdown of adenosine triphosphate (ATP) and plays an important role in the purinergic system. In the bladder, ATP is released from the urothelium in response to stretch, chemical irritation, and other stimuli, and downstream activation of purinergic receptors by ATP. Its breakdown products participate in mechanisms that modulate bladder sensory and motor functions [17]. Changes in purinergic signaling are known to contribute to bladder pathophysiology. Previous studies have shown that voided ATP levels are elevated in patients with OAB and detrusor overactivity [18]. In this context, the observed decrease in adenosine levels after BTX-A injections suggests that the therapeutic effects of BTX-A may result not only from direct action of BTX-A on the detrusor, but also from its impact on urothelial purinergic signaling, thereby decreasing sensory urgency and indirectly decreasing detrusor muscle overactivity.

Patients who responded to BTX-A treatment also displayed significantly increased urinary levels of N8-Acetylspermidine and guanidinoacetic acid compared with levels in patients without OAB symptom improvement. N8-Acetylspermidine is a polyamine derived from acetylation of spermidine. Polyamines are important regulators of cellular growth and differentiation, and also have immunosuppressive properties, whereby they can locally regulate inflammatory processes [19,20]. The urothelium capacity for fast regeneration and preservation of its structural and functional integrity is essential for proper bladder function. Urothelial cell shedding and breach of the urothelial barrier have been implicated in the development of urinary urgency and bladder overactivity due to an increase in activation of bladder afferent signaling and local inflammatory responses induced by direct contact of the underlying tissues with noxious urine contents [21]. In this context, the observed increase in N8-Acetylspermidine levels suggests possible effects of BTX-A treatment on urothelial cell turnover, urothelial regeneration, and reestablishing urothelial homeostasis, which would significantly contribute to OAB symptom improvement.

Guanidinoacetic acid, also known as guanidinoacetate, is involved in arginine metabolism. Arginine is an important amino acid involved in multiple metabolic pathways. One of these pathways involves breaking down arginine into urea and releasing nitric oxide (NO) [22]. In our study, we found elevated levels of guanidinoacetic acid after BTX-A injections which, considering it is an arginine substrate, indicates that arginine and its byproducts, such as NO, are elevated. NO released from urothelial and other cells has been proposed to modulate bladder function by either inhibiting or facilitating the activation of bladder afferent fibers [21]. An inhibitory effect for urothelial NO is supported by a recent study showing that intravesical treatment with NO-releasing nanoparticles ameliorated detrusor overactivity in an animal model [23]. These findings suggest another mechanism whereby BTX-A injections can improve OAB symptoms.

Overall, the changes in the urinary metabolome observed in this study suggest that mechanisms of BTX-A action on the bladder are much broader than its well-recognized inhibitory effect on detrusor contractility. Our findings indicate that improvement in OAB symptoms may also result from a two-pronged action of BTX-A on the bladder. This would involve repressing biological process that are detrimental while restoring those that are beneficial. As discussed above, the BTX-A impact on these processes is reflected by the significant reduction or increase in levels of metabolites linked to these biological processes.

From the 61 urinary metabolites analyzed, only 3 were significantly altered following BTX-A injections. These metabolites likely represent a small fraction of changes that occurred at the level of the bladder metabolome and would particularly reflect those occurring in the urothelium. Notably, all patients received BTX-A injections, but significant changes in the urinary metabolome following treatment only occurred in patients that reported improvement in their OAB symptoms. The failure of BTX-A treatment in some patients suggests that mechanisms underlying OAB symptoms in responders are different from those in non-responders. In this case, altered metabolites in responders following BTX-A may not only provide insights into the mechanisms of OAB pathophysiology, but can also assist in predicting treatment response.

Our study has some limitations, including the variable follow-up time points. Women were expected to follow up at 2 and 6 weeks post injection, but they often followed up at various time intervals ranging between 2 and 16 weeks. We were unable to control for bladder volume, diet, exercise, or home medications that may affect the bladder and urine composition. Considering, however, that each participant served as their own control (i.e., metabolite levels after BTX-A were calculated as the percent change from pretreatment levels), several of these factors would remain as they were before treatment, and minimally contribute to the observed post-treatment changes in the urinary metabolome. Additionally, urinary metabolite levels were normalized to the creatinine levels to account for urine dilution, sample volume effects, and kidney function. Another limitation is the relatively small sample size of our study, and therefore, we only had adequate statistical power to detect large differences (3- to 4-fold) across the groups. However, as in previous studies that enrolled similar numbers of OAB patients to conduct metabolomics analysis of urine and serum samples, significant information was generated [9,24]. Expanding our feasibility study to include a larger sample size would strengthen our findings and reduce variability in the data, which would not only improve statistical inferences, but could also potentially lead to identification of additional metabolites that are altered in patients with OAB who respond to BTX-A treatment.

This study is the first, to our knowledge, to evaluate changes in urinary metabolite levels after intravesical BTX-A injection for refractory OAB. Previous studies have focused on identifying urine and serum metabolites that could be used as biomarkers for OAB [9,24]. Our study has shifted this focus towards identifying unique urinary OAB metabolites that respond to OAB treatment, such as BTX-A injection. Future studies would entail also measuring changes in urinary metabolites before and after neuromodulation with the goal of identifying a urinary metabolome that “responds” to chemodenervation vs. neuromodulation. This would allow individualization of OAB treatment, instead of using a “one-size fits all” approach, and may demonstrate the unique pathophysiology of certain OAB phenotypes. This feasibility study demonstrates the potential and clinical impact of these future studies.

## 5. Conclusions

Intravesical BTX-A injection changes the urinary metabolome of patients with refractory OAB in conjunction with improvement in OAB symptoms. Metabolites that were altered following BTX-A can be linked to biological processes that may be involved in OAB pathophysiology and that reflect potentially novel mechanisms of BTX-A action on the bladder. Further investigation of urinary metabolites in larger patient samples and across various populations, including men and patients undergoing other treatment modalities for OAB, are expected to expand our findings and identify a urinary metabolome that can predict success after certain treatments, potentially allowing an individualized approach to OAB treatment.

## Figures and Tables

**Table 1:** Patient Demographics by improvement in OAB-V8 score after intravesical OnabotulinumtoxinA injection.

Characteristics	Total n = 21	No improvement n = 6	Mild n = 3	Moderate n = 6	Marked n = 6	*p* Value
Age (y), mean (SD)	56.8 (10.9)	55.2 (13.7)	51.33 (6.7)	54.83 (9.0)	63 (10.8)	0.41
BMI (kg/m^2^), mean (SD)	31.3 (4.9)	29.7 (5.7)	29.67 (3.5)	32.83 (4.6)	32.23 (5.4)	0.65
Race/Ethnicity, n (%)						0.23
Black	4 (19.1)	0	0	1 (16.7)	3 (50.0)	
Hispanic	16 (76.2)	5 (83.3)	3 (100)	5 (83.3)	3 (50.0)	
Other	1 (4.8)	1 (16.7)	0	0	0	
Diabetes, n (%)	3 (14.3)	2 (33.3)	0	1 (16.7)	0	0.35
Smoking history, n (%)	1 (4.8)	0	0	0	1 (16.7)	0.45
Previous pelvic surgery, n (%)	8 (38.1)	2 (33.3)	2 (66.7)	2 (33.3)	2 (33.3)	0.75

BMI, body mass index.

**Table 2:** Relative percent difference in metabolite levels during follow up when comparing the Mild, Moderate and Marked OAB-V8 score groups to the no-improvement in OAB-V8 score (None: our reference group).

Metabolite	Improvement in OAB-V8 Score ^a^			
	None [Reference]	Mild ß (95 % CI) ^§^	Moderate ß (95 % CI) ^§^	Marked ß (95 % CI) ^§^
**Adenosine**				
Unadjusted model	27.0	−127.0 (−251.42, −2.59) *	−127.0 (−239.54, −14.46) *	−37.0 (−132.11, 58.11)
Adjusted model ^b^	[Reference]	−123.74 (−229.16, −18.32) *	−106.91 (−206.24, −7.59) *	−23.09 (−120.49, 74.31)
**N-8 Acetylspermidine**				
Unadjusted model	−19.0	10.0 (−86.71, 106.71)	100.0 (14.71, 185.29) *	28.0 (−57.29, 113.29)
Adjusted model ^b^	[Reference]	3.03 (−89.81, 95.87)	124.23 (39.44, 209.02) **	56.0 (−35.84, 147.85)
**Guanidinoacetic acid**				
Unadjusted model	−4.0	30.0 (−53.79, 113.79)	91.0 (10.05, 171.95) *	71.0 (−9.95, 151.95)
Adjusted model ^b^	[Reference]	15.70 (−91.95, 123.34)	46.86 (−58.35, 152.09)	60.51 (−54.43, 175.45)

**a:** OAB-V8 score groups: None improvement < 8-points, Mild = 8–15 points, Moderate = 16–23 points, Marked ≥ 24-points. **b:** Median regression models were adjusted for age, BMI, diabetes, and race/ethnicity. **^§^** ß-Coefficients (and their corresponding 95% CI) indicates the percent difference in the average level of a urinary metabolite from an OAB-V8 group (e.g., “mild”) versus the “none” group. The coefficients represent percentage decrease or increase in metabolite levels when compared to the non-responders. The number in the None OAB-V8 reference group category is the estimated median percent change of metabolite from baseline to follow-up visit from the unadjusted median regression analysis. * *p* < 0.05; ** *p* < 0.01.

## Data Availability

The data that support the findings of this study are available from the corresponding authors upon reasonable request. Data are not publicly available to protect patient privacy.
